# Unveiling the Role of GRK2: From Immune Regulation to Cancer Therapeutics

**DOI:** 10.1155/mi/8837640

**Published:** 2025-03-05

**Authors:** Xizhuang Gao, Dehuai Jing, Yaowen Zhang, Fengqin Zhu, Yonghong Yang, Guangxi Zhou

**Affiliations:** ^1^Department of Gastroenterology, Affiliated Hospital of Jining Medical University, Jining Medical University, Jining 272000, Shandong, China; ^2^Medical Research Center, Affiliated Hospital of Jining Medical University, Jining 272000, Shandong, China

**Keywords:** autoimmune diseases, cancer, GRK2, immune cell, therapy

## Abstract

G protein-coupled receptors (GPCRs) represent humans' most prominent family of membrane proteins. In contrast, G protein-coupled receptor kinases (GRKs) play a pivotal role in the rapid desensitization of GPCRs. GRK2 is a particularly significant member of the GRK family. Recent studies have demonstrated that GRK2 primarily regulates immune cell function and homeostasis through receptor desensitization. Over the past decade, substantial progress has been made in elucidating the role of GRK2 in various human diseases. Notably, GRK2 is implicated in a range of autoimmune disorders, including rheumatoid arthritis (RA), inflammatory bowel disease (IBD), multiple sclerosis (MS), Sjögren's syndrome (SS), autoimmune myocarditis, hepatitis, and Graves' disease. Furthermore, emerging research has expanded our understanding of GRK2′s involvement in cancer biology. Comprehensive investigations into the biological and pathological functions of GRK2 have facilitated the development of therapeutic strategies aimed at targeting the GRK2 signaling pathway in cancer, inflammation, and autoimmune diseases. Promising results have been observed with targeted biologics in preclinical and clinical trials. This review aims to elucidate the multifaceted role of GRK2 in immune function, autoimmune diseases, and cancer to uncover the remaining complexities associated with this kinase. A thorough understanding of GRK2 may position it as a potent therapeutic target in treating inflammation and cancer.

## 1. Introduction

### 1.1. GRK2 and Autoimmune Diseases and Cancer

#### 1.1.1. Autoimmune Diseases and Cancer

The interaction between cancer and autoimmunity is intricate, with the underlying mechanisms remaining incompletely understood. Chronic inflammation constitutes a fundamental characteristic of autoimmune diseases, primarily resulting from dysregulation of the immune system, which leads to persistent inflammatory responses [1]. Recent studies suggest that chronic or excessive inflammation may contribute to the development of cancer, with inflammatory processes being implicated in over 20% of cancer cases. A substantial body of research has identified a strong association between specific autoimmune diseases and certain types of cancer. For instance, conditions such as Sjögren's syndrome (SS) and systemic lupus erythematosus (SLE) are associated with an elevated risk of malignancies [2]. Recent insights indicate that inflammation is involved in every stage of cancer development [3]. The interaction among cancer cells, the surrounding stroma, and inflammatory cells creates an inflammatory cancer microenvironment that promotes cancer initiation, growth, and transformation. Cells within this microenvironment exhibit high plasticity, enabling them to alter their phenotype and functions in response to environmental cues. Chronic inflammation and tissue damage resulting from autoimmune reactions can produce cytokines and chemokines that facilitate cancer development. In this context, inflammation can both induce cancer and precede its formation. Inflammatory mediators such as tumor necrosis factor–alpha (TNF-*α*), interleukin (IL)-6, transforming growth factor–beta (TGF-*β*), and IL-10 play significant roles in cancer development and progression [4].

#### 1.1.2. G Protein-Coupled Receptor Kinase (GRK) 2

**Figure 1:**
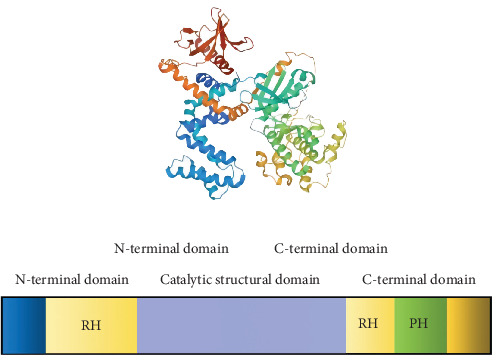
Structural organization and functional role of GRK2.

The G protein-coupled receptor (GPCR) family, comprising over 800 members, represents the most prominent group of membrane proteins in the human body. GPCRs play a pivotal role in various human diseases and serve as critical targets for drug development [5, 6]. GRKs rapidly desensitize GPCRs by phosphorylating the seven transmembrane domains [7]. GRK2 is a prominent member of the GPCR kinase family. GRK2 comprises a PH domain and a G protein signal transduction regulator homology (RH) domain [8]. GRK2 features a central catalytic domain, an N-terminal domain with the RH domain, and a C-terminal domain (Figure 1). These domains are crucial for regulating signal transduction and membrane localization. GRK2′s activity is regulated by interactions with proteins and lipids, including extracellular signal-regulated kinase (ERK), protein kinase A (PKA), protein kinase C (PKC), and G-protein beta-gamma (G*βγ*) [9]. GRK2 mediates the signal transduction, internalization, and sensitization of GPCRs. Evidence suggests that GRK2 is widely expressed in immune cells and contributes to the development of various diseases [10–15]. As a widely distributed protein, GRK2 regulates *β*-adrenergic receptors (*β*-ARs), angiotensin II type 1A receptors (AT1A-Rs), and chemokine receptors, which are crucial for vascular function, immunity, and inflammation. This discussion primarily focuses on regulating GRK2 in autoimmune diseases and cancer.

GRK2 is a critical regulator of GPCRs, the most prominent family of membrane proteins in humans. GRK2 comprises several key structural domains: the central catalytic domain, which phosphorylates the seven transmembrane domains of GPCRs, leading to their rapid desensitization; the N-terminal domain with RH domain, where the RH domain influences substrate specificity and modulates kinase activity by interacting with G*βγ* subunits; the C-terminal domain, which regulates GRK2′s kinase activity and its association with signaling pathways; and the pleckstrin homology (PH) domain, crucial for binding phosphoinositides, and facilitating GRK2′s membrane association and localization. GRK2 activity is further regulated by interactions with proteins and lipids, including ERK, PKA, PKC, and G*βγ*. This regulatory network is essential for GRK2′s roles in GPCR-mediated signal transduction, internalization, and sensitization. Figure 1 illustrates the structural features of GRK2 and emphasizes its significant role in modulating GPCR signaling pathways, highlighting its potential as a therapeutic target in various disease contexts.

## 2. GRK2 and Immune Cells

**Figure 2:**
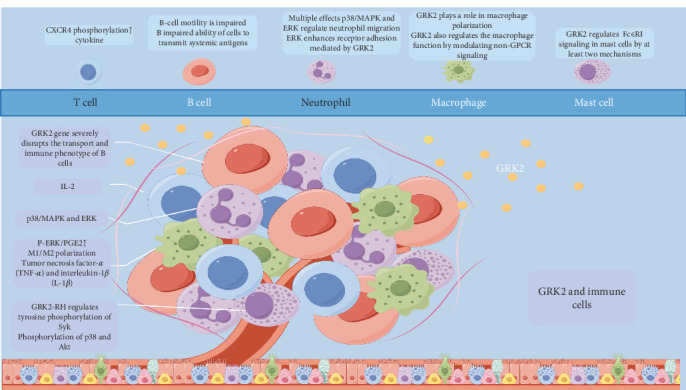
GRK2 is a key regulator of immune cell function, influencing various aspects of immune responses and cancer progression. This figure illustrates the role of GRK2 across different immune cell types, including T cells, B cells, neutrophils, macrophages, and mast cells. GRK2 modulates T cell receptor signaling, B cell motility, neutrophil migration, macrophage polarization, and mast cell degranulation, highlighting its pivotal role in maintaining immune balance. Dysregulation of GRK2 levels is implicated in autoimmune diseases and cancer, suggesting its potential as a therapeutic target for restoring immune system function.

Lymphocytes, which are key immune cells, are activated by antigen stimulation, triggering their proliferation and generating a targeted immune response [16]. Neutrophils and lymphocytes also contribute to immune responses mediated by macrophages, mast cells, and other cells [17]. GRK2 is extensively expressed across various immune cell types in response to cytokines (Figure 2).

### 2.1. T Cell

Under normal conditions, most T cells express the GRK2 protein, increasing its expression further during T cell mitosis [18]. After IL-2 stimulation, human T cells exhibit elevated mRNA and GRK2 protein levels [19]. GRK2 primarily regulates T cell receptor (TCR)-induced phosphorylation of CXCR4 and the formation of TCR-CXCR4 complexes, which promote cytokine production [20].

### 2.2. B Cell

Deletion of the GRK2 gene significantly disrupts B cell transport and immune phenotype, resulting in notably enlarged and deficient red marrow. Mice with GRK2 haploinsufficiency exhibit impaired B cell motility from the limbic zone to the follicle and reduced efficiency in delivering systemic antigens from B cells [21].

### 2.3. Neutrophils

Following bacterial infection, neutrophils gather at the site of infection. As the body's first line of defense, neutrophils can engulf and eliminate bacteria [22]. The combined effects of GRK2, p38/MAPK, and ERK regulate neutrophil migration, enabling them to reach infection sites and perform their bactericidal functions [23]. Inhibition of GRK2-mediated desensitization of formyl peptide receptor and phosphorylation by p38/MAPK promotes neutrophil migration. Conversely, ERK enhances GRK2-mediated receptor adhesion [23]. Our subsequent research should focus on resolving this paradox.

### 2.4. Macrophages

Inflammatory and cancerous tissues predominantly contain M1-type macrophages. GRK2 influences macrophage polarization under diverse conditions [24]. CGP4212 activates the AT2A-R by binding to phosphorylated ERK1/2 (p-ERK1/2), promoting GRK2′s translocation to the cytoplasm. This process inhibits the complete phosphorylation of the I*κ*B inhibitor (I-B) and disrupts NF-*κ*B signaling, resulting in reversible macrophage polarization in synovial tissues and subsequent fat storage [25]. We have reported that the deletion of GRK2 reprograms macrophages to an anti-inflammatory phenotype by restoring GPCR signaling. Therefore, in a mouse model of collagen-induced arthritis, we genetically degrade GRK2 using GRK2^f/f^Lyz2-Cre^+/−^ mice. Synovial inflammation and M1 polarization were improved in GRK2^f/f^Lyz2-Cre^+/−^ mice [26]. In a model of dextran sodium sulfate (DSS)–induced colitis in GRK2 heterozygous mice, we found that prostaglandin E2 (PGE2) stimulation in the lamina propria of the colon could supersensitize EP4 receptors on monocytes. This hypersensitivity, in combination with cAMP and cAMP-responsive element-binding proteins, promotes M2 polarization through a mediated mechanism, revealing for the first time the dysregulation of the M1/M2 macrophage ratio in ulcerative colitis (UC). Further experiments demonstrated that GRK2 affects macrophage polarization by decreasing cAMP levels and inhibiting the cAMP response element-binding (CREB) pathway, leading to reduced M2 polarization in UC [27].

GRK2 additionally influences macrophage functionality by altering non-GPCR signaling pathways. Macrophages primarily secrete inflammatory cytokines, including TNF-*α* and IL-1*β*. A previous study demonstrated a negative correlation between GRK2 levels, activity, and p38/MAPK levels. Furthermore, LPS-induced mitochondrial translocation of GRK2 reduces the production of reactive oxygen species (ROS) and proinflammatory cytokines [28]. In GRK2^^+/−^ mice, peritoneal macrophages are stimulated with LPS phosphorylate p38/MAPK and release TNF-*α*. In LysM-GRK2^^+/−^ mice, GRK2 knockdown markedly decreases LPS-induced production of inflammatory cytokines in macrophages [29].

GRK2 plays an important role in macrophage polarization and function, primarily influencing the presence of M1-type macrophages. In inflammatory and cancerous tissues, GRK2 promotes M1 polarization by regulating various signaling pathways but can also be transformed into an anti-inflammatory M2 phenotype through genetic deletion. For example, in a model of DSS-induced colitis, PGE2 stimulation leads to hypersensitivity of EP4 receptors that bind cAMP and CREB proteins, promoting M2 polarization. This reveals a dysregulated M1/M2 ratio in UC. Additionally, GRK2 regulates the ability of macrophages to secrete inflammatory cytokines through non-GPCR pathways, thereby affecting their function and inflammatory response.

### 2.5. Mast Cells

Mast cells, which are nonproliferative and long-lived, reside in specific tissues and play a vital role in innate and adaptive immunity. Reducing GRK2 expression by half resulted in a significant decrease in antigen-induced calcium mobilization and degranulation, completely halting the production of cytokines IL-6 and IL-13. The influence of GRK2 on cytokine production operates through the phosphorylation of p38 and Akt without requiring its catalytic activity. Increased expression of GRK2 or its RH structural domain (GRK2-RH) amplifies antigen-induced Ca^2+^ mobilization, thereby enhancing mast cell degranulation and cytokine production without altering the levels of any Fc*ε*RI subunits (*α* and *γ*). This overexpression does not affect antigen-induced phosphorylation of Fc*ε*RI *γ* or Src but enhances the tyrosine phosphorylation of spleen tyrosine kinase (Syk). These findings indicate that GRK2 modulates Fc*ε*RI signaling in mast cells through two distinct pathways: one involving the GRK2-RH domain, which affects Syk's tyrosine phosphorylation, and the other through the phosphorylation of p38 and Akt [30, 31].

The observations underscore the pivotal role of GRK2 in regulating the immune system's functionality across various cell types, including lymphocytes, neutrophils, macrophages, and mast cells. Given its broad regulatory impact on cell migration, cytokine production, and antigen response, GRK2 is crucial for maintaining immune balance. Dysregulation of GRK2 levels has been implicated in the pathology of autoimmune diseases and cancer, suggesting that it plays a significant role in immune tolerance, inflammation, and the immune system's ability to combat malignancy. Consequently, exploring the functional implications of altered GRK2 levels in these diseases offers a promising avenue for identifying novel therapeutic targets. Understanding how GRK2 dysregulation contributes to disease mechanisms can guide the development of targeted interventions to restore immune system balance and function. This approach can potentially advance the treatment of autoimmune conditions and cancer, highlighting the importance of further research into GRK2′s role in immune regulation and disease pathogenesis.

## 3. GRK2 and Autoimmune Diseases

**Figure 3:**
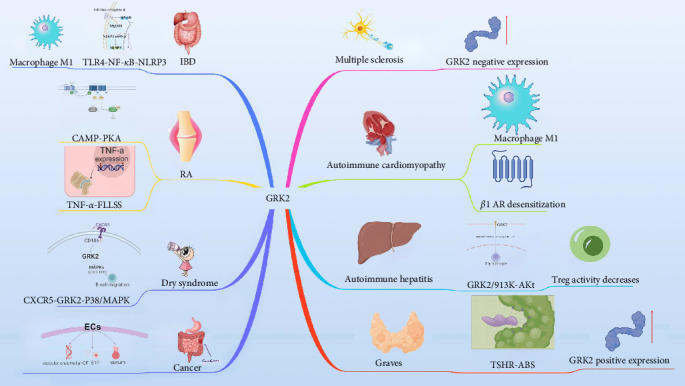
Influence of GRK2 on immune diseases and cancer.

Numerous immune diseases are closely associated with the pathogenesis of GRK2, which regulates immune function (Figure 3), including inflammatory bowel disease (IBD), rheumatoid arthritis (RA), SS, and multiple sclerosis (MS), autoimmune cardiomyopathy, autoimmune hepatitis (AIH), Graves, etc. GRK2 is thought to be a popular target for these diseases [32, 33]. This review aims to examine the biological processes involved in GRK2 in autoimmune diseases and identify a novel therapeutic target for treating autoimmune diseases.

Figure 1 illustrates the role of GRK2 in several immune diseases, including RA, IBD, primary Sjögren's syndrome (PSS), MS, autoimmune cardiomyopathy, AIH, and Graves' disease. In RA, GRK2 promotes the proliferation and EP4 receptor desensitization of fibroblast-like synovial (FLS) cells, contributing to joint damage. In IBD, it mediates the inflammatory response via the TLR4-NF-*κ*B-NLRP3 pathway. GRK2 enhances B-cell migration in PSS, while in MS, lower GRK2 expression in PBMCs suggests its potential as a biomarker. Additionally, GRK2 drives M1 macrophage polarization and *β*1-AR desensitization in autoimmune cardiomyopathy, leading to cardiomyocyte apoptosis. In AIH, it regulates Treg cell function through the PI3K-Akt pathway, and in Graves' disease, GRK2 activity is increased by TSH receptor (TSHR)–specific autoantibodies, exacerbating the autoimmune response. The figure highlights GRK2′s critical involvement in immune modulation and its potential as a therapeutic target in autoimmune diseases.

### 3.1. GRK2 and RA

Chronic autoimmune diseases such as RA are common. The main pathological features include synovial hyperplasia, vascular opacity, cartilage damage, and bone erosion [34]. Pathophysiologically, the disease arises from the abnormal proliferation of FLS cells. Approximately two-thirds of the proliferating synovium comprises FLS, which secrete adhesion molecules and inflammatory cytokines that damage cartilage and bone. RA polyarthritis is caused by the migration of FLS to unaffected cartilage. Enhanced GRK2 translocation and PGE2 receptor (EP4) desensitization play essential roles in FLS dysfunction [35]. Furthermore, FLS and endothelial cells (ECs) express and activate GRK2. The PGE2-cAMP-PKA signaling pathway promotes GRK2 translocation to the cell membrane and activates ERK1/2 during RA, leading to EC migration and synovial angiogenesis [36]. Conversely, GRK2 encourages EP4 receptor hypersensitization and reduces cAMP production in FLS abnormalities [37]. Recent studies have shown that TNF-*α* stimulates FLS; TNF-*α* receptor 2 transactivates GRK2 via TRAF2, promoting desensitization of EP4 receptors, which leads to cancer-like proliferation of FLS and contributes to disease progression in RA [38].

GRK2 expression varies among cells during RA [39]. The expression of GRK2 is reduced in T helper (TH) and B cells of arthritic rats but not in CD8+ T cells [40]. It has been found that peripheral blood mononuclear cells (PBMCs) from RA patients contain reduced levels of GRK2 activity and protein [41]. Additionally, GRK2 expression is decreased by ~50% in lymphocytes of RA patients, resulting in diminished GRK2-mediated chemokine receptor desensitization and enhanced T-cell chemotaxis to CCl4 [12]. In light of the differing functional expressions of GRK2 in RA, current research focuses on whether GRK2 can be targeted as an alternative treatment, with GRK2 inhibitors being considered to restore immune homeostasis [42].

### 3.2. GRK2 and IBD

An abnormal immune response to intestinal and colonic flora primarily causes IBD [43]. Despite this, the exact pathogenesis of the disease remains unknown. IBD mainly affects immune cells that process and deliver antigens, activating adaptive immunity. Activating these immune cells can also produce inflammatory mediators, such as cytokines and chemokines, which help enhance the immune response in the lining of the gastrointestinal tract [44]. Inflammasomes mediate the inflammatory response and are considered essential regulators of IBD. Recent studies indicate that inflammatory tissue activation plays a critical role in the pathogenesis of IBD [45]. Inflammasomes can be formed by the NOD-like receptor family, particularly the pyrin domain-containing 3 (NLRP3), one of the best-studied NOD receptors. When inflammatory cells become activated, NF-*κ*B initiates the transcription of components such as NLRP3 and pro-IL-1*β* [46–50].

There is evidence that GRK2 is involved in the pathogenesis of IBD [51, 52]. In a mouse model of DSS-induced colitis, it has been shown that GRK2 is involved in inflammatory gene transcription in DSS-treated mice. In response to the DSS challenge, heterozygous knockdown of GRK2 or myeloid-specific depletion of GRK2 significantly reduced the severity of IBD and colitis [51]. In mice with UC, GRK2 activation mediates the TLR4-NF-*κ*B-NLRP3 inflammatory pathway, promotes macrophage M1 polarization, increases the expression of inflammatory cytokines (i.e., TNF-*α*, IL-1*β*, IL-6, IL-12), and thereby compromises the intestinal mucosal barrier [7, 52]. Given that GRK2 is crucial in mediating intestinal inflammation, drugs targeting GRK2 may offer a potential treatment for IBD.

### 3.3. GRK2 and Primary Dry Syndrome With MS

PSS is a chronic inflammatory autoimmune disease characterized by exocrine gland dysfunction [53, 54]. A hallmark of PSS is abnormal B-cell function, and activated CXCR5-GRK2-p38/MAPK signaling enhances B-cell glandular migration in PSS mice, contributing to disease progression [55].

Chronic inflammatory demyelinating central nervous system diseases are referred to as MS. A significant reduction in GRK2 expression has been observed in PBMCs from MS patients [56, 57]. Moreover, GRK2 expression was negatively correlated with MS disease activity. Studies using mouse models have demonstrated that GRK2^+/−^ mice exhibit more severe symptoms during the relapsing-remitting phase of MS [58].

In summary, this study elucidates the distinct roles of GRK2 in the pathogenesis of PSS and MS. In the case of PSS, GRK2 significantly contributes to disease progression by facilitating the migration of B cells to epithelial tissues, providing new insights into the pathological mechanisms underlying PSS. Concurrently, in the context of MS, a notable reduction in GRK2 expression was observed, which inversely correlates with disease activity. This finding suggests the potential of GRK2 as a biomarker for assessing the severity of MS. However, further clinical studies and experimental validations are imperative to fully understand the specific mechanisms of GRK2′s action in these diseases and its viability as a biomarker. This research underscores the importance of GRK2 in the pathophysiology of both PSS and MS, opening new avenues for future research, particularly in developing diagnostic and therapeutic strategies for these conditions.

### 3.4. GRK2 and Autoimmune Cardiomyopathy

Autoimmune myocarditis is a complex inflammatory response characterized by clinical and histological manifestations resulting from an abnormal immune process [59]. In the cardiomyocytes of mice with experimental autoimmune myocarditis, immune cells were in sync with the immune response. Each cell subtype exhibited different pathway activities in the myocarditis model, suggesting that these cells play distinct immunological roles in various biological pathways. Notably, macrophages and TH17 cells are markers of the inflammatory phase [60]. The M1 polarization of macrophages primarily releases inflammatory factors and increases the degree of inflammatory infiltration. In autoimmune myocarditis, macrophages are polarized toward the M1 phenotype, and GRK2 expression is upregulated [61].

Dilated cardiomyopathy (DCM) can progress from autoimmune myocarditis [62]. The G*βγ* pathway has been shown to interact with GRK2 to induce chronic *β*-AR desensitization, leading to heart failure (HF) [63, 64]. Immunomodulatory disorders in the DCM heart primarily include altered cytokine levels [65], autoantibodies against various cardiac proteins [66, 67], T-lymphocyte subsets [68], and cell-mediated inflammation [69]. Autoantibodies targeting the second extracellular loop (ECII) of the *β*1AR are the primary autoimmune targets in patients with DCM [66, 70, 71]. In addition to inducing positive inotropic responses and apoptosis in isolated cardiomyocytes, monoclonal antibodies against the ECII of the *β*1AR also promote apoptosis in these cells [72, 73]. Recent studies have shown that both *β*1AR ECII fusion protein-immunized rats and *β*1AR DNA-immunized mice exhibit impaired cardiac function [74, 75] and an increase in *β*1-AR kinase mRNA, along with the upregulation of GRK2, is the first step in desensitizing the *β*1AR.

In conclusion, GRK2 promotes the polarization of M1 macrophages in autoimmune myocarditis and is involved in the desensitization of the *β*1AR in DCM, leading to apoptosis of cardiomyocytes.

### 3.5. GRK2 and AIH

AIH, an inflammatory liver disease, is primarily driven by a T-cell-mediated autoimmune response [76]. AIH can, therefore, be considered a T-cell disease [77, 78]. T-cell expression significantly differed between patients with AIH and those without the condition [79]. In addition, the defective immunomodulation of AIH may result from reduced regulatory T cell number and function, and cellular glycolipid metabolism is one of the critical factors affecting Treg differentiation [80, 81].

GRK2 is critical for regulating phosphorylation-dependent GPCR desensitization, endocytosis, intracellular translocation, resensitization, and subsequent intracellular signaling pathways. Recent studies indicate that GRK2 interacts with PI3K, Akt, and MEK, implicating its involvement in inflammation, cardiovascular disease (CVD), and cancer therapeutics [18, 82]. Among these interactions are the GRK2/PI3K-Akt pathway, which regulate immune cell metabolism. Through its interaction with PI3K, GRK2 promotes the recruitment of PI3K to the cell membrane and participates in signal transduction [83]. In a mouse model of immune hepatitis, coexpression of GRK2 with PI3K regulates T lymphocyte lipid metabolism, reduces Treg activity, and leads to immunodeficiency in immune hepatitis [84].

### 3.6. GRK2 and Graves

Graves' disease is an autoimmune disorder in which the thyroid gland becomes overactive due to the action of TSHR-specific conformation-dependent autoantibodies. Ligands bound to the TSHR trigger its coupling to G proteins [85–89]. Thus, the agonist attaching to TSHR activates G proteins, leading to receptor phosphorylation via GRKs [90, 91]. It has been demonstrated that c-TSHR autoantibodies (c-TSHR-Abs), while not activating G protein-dependent signaling, can activate GRK2 and downstream signaling pathways, resulting in the accumulation of intracellular inclusion bodies, ROS production, and cell death, which exacerbates the severity of Graves' disease [92]. Therefore, GRK2 activity is enhanced in Graves' disease [93].

## 4. GRK2 and Cancer

In the regulation of cancer, GRK2 accumulates in the presence of DNA-damaging agents (e.g., adriamycin) that activate cell cycle arrest, helping to counteract the stimulation of the p53 pathway induced by the G2/M checkpoint mechanism and preventing the apoptosis of blocked cells [94]. When TGF-*β* stimulates the ALK5 receptor, GRK2 binds to and phosphorylates Smad2/3, preventing the nuclear translocation of the Smad complex, thereby inhibiting the proapoptotic effect of TGF-*β* and potentially enhancing its anticancer effects [95]. GPCRs associated with cancer activate MAPK and promote cell proliferation. As a result of its association with GIT-1, GRK2 promotes lipid-sensing S1P1 MAPK stimulation in epithelial cells, whereas it promotes beta-arrestin (betaARR) MAPK activation in astrocytes [96, 97]. In fibroblast cell lines, GRK2 enhances hedgehog (HH)/smoothened (SMO)-mediated transformation [98]. Our research revealed tight HH pathway regulation during tumor development. In this context, SMO is derepressed and requires the presence of betaARR2 and GRK2 for the activation of the glioma-associated oncogene (GLI) signaling, ultimately driving the transcription of the downstream HH-related gene 8E14 [99]. Gli1 protein expression in colorectal cancer tissue correlates with several leading-edge cancer clusters, infiltration depth, lymph node metastasis, and the TNM stage of colorectal cancer. As a transcriptional activator, Gli1 plays a crucial role in the HH pathway [100, 101]. In colorectal cancer tissues, Gli1 is coexpressed with cancer stem cell (CSC) markers, including SOX9 and CD133. Inhibition of GLI-1 expression reduced the expression of these markers in gastric cancer cells, suggesting an essential role for GLI-1 in colorectal cancer [102, 103]. Based on this information, we can speculate that GRK2 affects GLI1 protein expression in the HH signaling pathway, participating in the regulation of colorectal cancer.

Furthermore, within the cancer microvascular environment, the endothelial-specific downregulation of GRK2 appears to promote the recruitment of macrophages to cancer sites directly through altered chemotactic secretion and indirectly through the promotion of leaky blood vessels. When GRK2 is downregulated, the response of ECs to relevant angiogenic stimuli (vascular endothelial growth factor [VEGF], sphingosine-1-phosphate [S1P], serum) is enhanced, altering the ability of these cells to organize into tubular structures and disturbing the balance between inflammation and the secretion of angiogenic factors. Additionally, reduced levels of GRK2 affect the TGF-*β* signaling pathway, which regulates angiogenesis's activation and catabolic phases through timely modulation of the opposing effects of the ALK1 and ALK5 receptors [104, 105].

### 4.1. The Role of GRK2 in Tumor Progression Can Be Summarized as Given Below

#### 4.1.1. Regulation of Cell Proliferation and Tumor Growth

GRK2 inhibition reduces cancer cell proliferation and tumor growth by suppressing G2/M cell cycle progression and activating immune cells [106].

#### 4.1.2. Key Role in Multiple Cancers

GRK2 plays a role in the molecular pathophysiology of various cancers by regulating various molecular processes. It plays a crucial role in lymphocyte responses to factors such as chemokines and leukotrienes, which may be related to the immune environment of tumors [18].

#### 4.1.3. Different Roles in Specific Cancer Types

GRK2 reduces cell proliferation in thyroid cancer, suggesting a possible inhibitory role in some cancer types [107]. However, in breast cancer and other cancer types, GRK2 promotes growth factor signaling and cell proliferation, suggesting a possible promotional role in these cancers [108].

GRK2 plays an important role in physiological and tumor angiogenesis, regulating EC activation and migration. GRK2 downregulation enhances the response of MLECs to S1P and VEGF, promoting cell migration and PDGF-BB secretion. However, in retinal vascular development, GRK2 deficiency leads to defects in tip cell protruding foot formation, reducing vessel expansion. Overall, the role of GRK2 is complex and influenced by environmental signals [104].

GRK2 is critical in tumor progression, exhibiting diverse effects across cancer types. It modulates cell proliferation and tumor growth, demonstrating contrasting roles in specific cancers, such as thyroid and breast cancer, and is integral to tumor angiogenesis. This underscores its potential as a significant target in cancer therapy.

## 5. GRK2 as a Potential Therapeutic Target

GRK2 dysfunction is associated with cancer and autoimmune diseases, making it a potential therapeutic target. Therefore, it is essential to develop GRK2-specific substrates and inhibitors for studying GRK2-mediated cellular functions and to create drugs that target GRK2 directly.

### 5.1. GRK2 and Inhibitors

GRK2, a vital regulator of the inflammatory response, exhibits altered expression and function under various inflammatory conditions. It has been implicated as an essential protein in the pathogenesis and progression of related diseases. GRK2 inhibitors, such as balance, Takeda's paroxetine, and its derivatives, M119, and gallein, have different structures and inhibitory effects on their respective conditions. The ATP mimetic balanol is a metabolite produced by Verticillium balanoides that acts as a competitive inhibitor of ATP in GRK2′s kinase domain [109]. Due to its heterocyclic structure, balanol possesses a lower affinity for GRK2 than Takeda inhibitors, which inhibit the enzyme by inducing a slight closure of GRK2′s structural domain [110]. Paroxetine, a selective serotonin reuptake inhibitor (SSRI), binds specifically to the kinase structural domain of GRK2 with off-target affinity and selectively inhibits GRK2 kinase activity in the micromolar range [111]. The structure of GSK180736A is similar to that of paroxetine, making it a derivative. In addition to cocrystallizing at GRK2′s active site, GSK180736A is also identified as an enzyme inhibitor [112]. These inhibitors suggest GRK2 could be a promising new drug target for treating these diseases.

### 5.2. GRK2 and Peptide Substrates

Developing highly selective and sensitive GRK2 peptide substrates will enhance our understanding of GRK2-mediated cell signaling mechanisms and aid in the design of drugs targeting GRK2. Inhibitors targeting a wide range of protein–protein interactions may be developed from short peptide substrates of GRK2. Such inhibitors could serve as promising therapeutic candidates for various diseases and tools for regulating intracellular signaling pathways. However, no practical method for measuring GRK2 activity in live cells exists. In contrast to full-length natural protein substrates, short synthetic peptides are more stable, easier to purify, more convenient to manufacture, and more straightforward to store. In our study, we identified a peptide substrate for GRK2 that is highly selective and exhibits a high affinity for microtubulin (GR-11-1), with a significantly lower affinity for any other protein substrate evaluated [113]. We have demonstrated that peptides can be used to develop artificial biosensors for detecting hyperactivated enzymes in diseased cells [114].

Additionally, peptide substrates can be utilized to create molecular tools that act as molecular antennas on the surface of nanoparticulate formulations (e.g., for targeting, membrane permeation, receptor ligation) or as components of fluorescent biological sensors (e.g., by phosphorylation followed by changes in fluorescence intensity; phosphorus/Thr recognition) [115, 116]. Recent advances in drug delivery systems, based on protein transduction domains and cell-penetrating peptides that modify nanoparticle preparations, offer new methods for introducing biomolecules into living cells [117]. We have found that introducing specific peptide substrates for target kinases into living cells is a promising approach for developing diagnostic methods, therapeutic procedures, and assays for kinase activity [114]. The intracellular delivery of GRK2-specific peptide substrates may provide new opportunities for basic research into GRK2 and its biological roles in diagnosing and treating GRK2-related diseases, such as cancer and autoimmune disorders.

## 6. Conclusion

In summary, this review elucidates the pivotal role of GRK2 in the pathophysiology of autoimmune diseases and malignancies. GRK2 is a critical regulator of immune cell signaling, influencing the functional dynamics of various immune cell populations and their interactions within the tumor microenvironment. Dysregulation of GRK2 has been implicated in the etiology of several autoimmune disorders, including RA, IBD, and MS, where it disrupts immune homeostasis and promotes pathological inflammation. Furthermore, GRK2′s involvement in cancer biology is underscored by its capacity to modulate tumorigenesis, metastasis, and angiogenesis through intricate signaling networks.

Given its dual role in immune regulation and tumor biology, GRK2 emerges as a promising therapeutic target. Future investigations should prioritize the development of selective GRK2 inhibitors and peptide substrates, which may provide novel insights into its mechanistic pathways and therapeutic potential. A multidisciplinary approach that integrates molecular biology, pharmacology, and clinical research will be essential to fully elucidate GRK2′s contributions to immune dysregulation and cancer progression. Ultimately, advancing our understanding of GRK2 may facilitate the development of innovative therapeutic strategies aimed at restoring immune equilibrium and enhancing antitumor immunity.

## Data Availability

Data availability is not applicable to this article as no new data were created or analyzed in this study.
